# A qualitative exploration of the perspectives of mental health professionals on stigma and discrimination of mental illness in Malaysia

**DOI:** 10.1186/s13033-015-0002-1

**Published:** 2015-03-10

**Authors:** Ainul Nadhirah Hanafiah, Tine Van Bortel

**Affiliations:** Cambridge Institute of Public Health, Department of Public Health and Primary Care, University of Cambridge School of Clinical Medicine, Box 113, Cambridge Biomedical Campus, Forvie Site, Robinson Way, Cambridge, CB2 0SR UK

**Keywords:** Mental health, Malaysia, Mental health Malaysia, Mental health professionals, Mental illness, Stigma, Discrimination

## Abstract

**Background:**

Stigma of mental illness has been identified as a significant barrier to help-seeking and care. Basic knowledge of mental illness - such as its nature, symptoms and impact - are neglected, leaving room for misunderstandings on mental health and ‘stigma’. Numerous researches have been conducted on stigma and discrimination of people with mental disorders. However, most of the literature investigates stigma from a cultural conception point of view, experiences of patients or public attitudes towards mental illness but little to none from the standpoint of mental health professionals. In Malaysia, this research on stigma is particularly limited. Therefore, the state of stigma and discrimination of people with mental illness was investigated from the perspectives of mental health professionals in Malaysia.

**Methods:**

In-depth, face-to-face, semi-structured interviews were conducted with 15 mental health professionals from both government and private sectors including psychiatrists, psychologists and counsellors. The interviews were approximately 45-minutes long. The data was subsequently analysed using the basic thematic approach.

**Results:**

Seven principal themes, each with their own sub-themes, emerged from the analysis of ‘stigma of mental illness’ from mental health professionals’ point of view, including: (1) main perpetrators, (2) types of mental illness carrying stigma, (3) demography and geography of stigma, (4) manifestations of stigma, (5) impacts of stigma, (6) causes of stigma and (7) proposed initiatives to tackle stigma. Stigma of mental illness is widespread in Malaysia. This is most evident amongst people suffering from conditions such as schizophrenia, bipolar disorder and depression. Stigma manifests itself most often in forms of labelling, rejection, social exclusion and in employment. Family, friends and workplace staff are reported to be the main perpetrators of discriminatory conducts.

**Conclusion:**

According to the perspectives of the mental health professionals, implications of stigma include patients being trapped in a vicious cycle of discrimination leading to detrimental consequences for the individual, their families, communities and society as a whole. There is a pressing need to address stigma of mental illness and its consequences, especially through raising awareness of mental health and wellbeing in Malaysia, as reported by the mental health professionals.

## Introduction

Individuals with mental illness often struggle a double-edged sword battle. Coping with the symptoms of the condition itself is already difficult enough whilst misperceptions of the condition create further complications such as suffering negative connotations - ‘stigma’ - and discrimination [1-3]. Stigma and discrimination are also suggested to be significant barriers to mental health help-seeking, mental health recovery and social inclusion [4-7].

### Global stigma and discrimination of mental illness

Stigma and discrimination is not limited to mental illness. Physical medical conditions such as HIV/AIDS [8] and obesity [8-10] often face similar challenges [11]. However, in comparison, people with mental health problems suffer higher stigmatisation and discrimination in several areas of their life such as in social relationships and employment [9,10]. Likewise, civil society is often inclined to hold people with mental disorders responsible and accountable for their condition and are less sympathetic to them [12]. These negative attitudes often result in discriminatory behaviours. Thornicroft et al. [3] encapsulated stigma of mental illness as arising from the following three issues:

- Problems of knowledge – ignorance;
- Problems of attitudes – prejudice;
- Problems of behaviour – discrimination.

The presence of stigma of mental illness has consistently demonstrated to be a significant debilitating factor towards people with mental health problems. It creates the possibility for a vicious cycle of discrimination and worsening condition. This in turn reinforces the stigma and causes disadvantages for people with mental health problems in various aspects of life and its opportunities such as in social relationships, employment and health recovery [2,13,14].

A large volume of previous studies on stigma of mental illness implicated that it occurs more often in Western societies [15,16]. However, more recently, the World Health Organisation (WHO) suggested that stigma of mental illness equally affects Western and Asian communities alike [17]. This notion is supported by results of Fabrega [18], Lauber and Rossler [19], and Ng [20] whereby each study indicated prevalent stigma amongst Asian communities in India, China and Malaysia. This study investigated perspectives on stigma and discrimination of mental illness from the point of view of mental health professionals in Malaysia.

### Review of stigma studies in Malaysia

In the past decade, concurrent with the development of stigma of mental illness research in Western counterparts, studies exclusive to Malaysian society have gradually increased. Nevertheless, earlier researches mostly focused on conceptual understanding of stigma [18-20]. Therefore, recent research acted in response to the pressing need for studies on the experiences of people with mental health problems [21-24]. Mubarak et al. [24] and Khan et al. [22] each conducted studies related to depression and/or schizophrenia and/or depression whereby the former study focused on the experiences of schizophrenia patients and the latter investigated public perceptions towards people with depression and schizophrenia. Results from Khan et al. [22] indicated manifestations of stigma towards those with the mental disorders which can result in negative consequences such as social rejection and unfair blame of the cause of mental illness to the bearer. Unsurprisingly, Mubarak et al. [24] found that approximately one third of patients were dissatisfied with their overall quality of life and general wellbeing and at least half of the patients reported an adverse impact on their lives, financial independence, social relationships and employment. In fact, those employed described their experience of significant exploitation in the workplace.

Similar results were reported in three global studies conducted by Thornicroft et al. [25], Lasalvia et al. [26,27] and Rose et al. [28] in which Malaysia took part as a study site. They reported that, regardless of country, experienced discrimination is predominant amongst persons with schizophrenia and depression across all study sites. Participants also reported social exclusion, discrimination by family members and limited job opportunities to be major issues. These studies provide awareness regarding perceptions leading to stigma and discrimination but also its impact and the measures that can be taken to tackle the issue.

Acknowledging the overarching and far-reaching impacts of stigma and discrimination of ill mental health [29,30], Chang and Horrocks [21] were interested to investigate these in relation to perceptions of stigma of ill mental health in Chinese families. Their study was aimed at informal caregivers’ experiences with relatives with severe and persistent mental disorders. All participants believed that patients are unable to care for themselves, that they are unpredictable and possibly aggressive. When faced with observable behaviours, civil society may be inclined to label, stigmatise and discriminate against people with mental health problems. It appeared that due to stigma, families avoided discussing the patients’ illness with others including extended family and friends. Comparable to studies of Fabrega [18] and Ng [20], the families perceived mental illness as shameful and thus avoided disclosure in order to protect the family from ‘losing face’. Education of both the families and the wider public were suggested to be crucial in reducing stigma, as negative perceptions and behaviours stem from erroneous understanding of mental health.

Minas et al. [23] aimed to investigate these two notions within the Malaysian context by comparing attitudes of hospital staff towards mental illness and diabetes. The results of the between-group design indicated differences in attitudes and expected behaviour amongst the healthcare staff. Reports illustrated stigmatising attitudes towards patients with mental health problems as high. This correlated to lower tendency for care and support with higher inclination of avoidance and expected negative stereotype as compared to diabetic patients. Consequences of such stigma and discrimination outcomes are debilitating as it may impact on the quality of services delivered. As such, this implies not only the urgency to examine stigma from the perspectives of patients and the public but also from the perspectives of mental health professionals and other service providers.

In light of the attention towards mental health, Jamaiyah [31] argued integration of mental health into primary care requires a lot more attention and improvement. This has been supported through reviews by Mubarak [32] and Haque [33]. Concerns related to mental health and its services include low awareness, poor regulation especially within non-medical professionals as well as the lack of advocacy and support from groups other than Government bodies. Particularly, despite increased numbers of mental health research in Malaysia, the gap within knowledge and practice still persists. This causes insufficiency to stimulate and establish significant impact or changes within the healthcare system.

### Rationale of present study

Whilst the abovementioned studies have contributed towards the current global stigma literature, several improvements could be made. Firstly, with mental health professionals being crucial members to mental health initiatives, insights from this stakeholder group are equally pivotal for ensuring sustainable development of mental health services. However, studies examining the perspectives of mental health professionals are scarce in Malaysia. Secondly, understanding stigma from an integrated point of view is important to ensure that the needs of all stakeholder groups are taken into account. The literatures reviewed above were conducted in relation to specific conditions such as schizophrenia and may not be representative of other types of mental health conditions. Finally, literatures within the Asian context are being overshadowed by Western studies. Therefore, more culturally sensitive and contextual research is much needed in order to reflect the specific circumstances of each locality in the global combat against stigma and discrimination of people with mental health problems.

### Aim of present study

This study aims to contribute towards bridging the current gap in research on stigma and discrimination of mental illness in Malaysia. Current literatures lack insight from mental health professionals thus the state of stigma towards people with mental illness was explored from the perspectives of mental health professionals. This was conducted within urban settings in Malaysia.

## Methods

This research formed part of the first author’s Masters programme and therefore the first author assumed the role of the main researcher. Ethical approval (PNM/11/12-73) from King’s College London Psychiatry, Nursing and Midwifery Research Ethics Subcommittees, PNM RESC, was obtained prior to commencement of this project. Primary data collection for this study took place in Malaysia between April and July 2012. Face-to-face semi-structured interviews with 15 mental health professionals were carried out in Kuala Lumpur and Selangor, two urban states in West Malaysia. This sample comprised of six females and nine males aged between 35–65 with educational and training backgrounds as psychiatrists, clinical psychologists or counsellors both from government and private institutions providing services such as psychiatric treatment, psychological therapy and counselling.

Inclusion criteria for this study required the interviewed participant be a trained mental health professional who currently actively worked with patients in either government or private mental health care practices. In this context, ‘private mental health care practice’ refers to any organisations, clinics or hospitals that are not government-linked or owned. Additionally, they must already be in practice for more than 5 years and able to understand and converse fluently in English as the interviews were fully conducted in English. Due to the many languages spoken in Malaysia, English was the language of choice in this study as it is the universally spoken and written language across the country.

The study consisted of 15 participants, 5 of whom were government psychiatrists. The remaining 10 were private health care providers that including 2 counsellors, 3 psychiatrists and 5 clinical psychologists. All participants were sampled within an urban setting of Kuala Lumpur and Selangor, two major cities in Malaysia.

All government professionals were recruited from the Department of Psychiatry at the General Hospital Kuala Lumpur which provides most of the comprehensive psychiatric care in Malaysia. Approval was first obtained from the Director of the department and the Director’s assistant shared contact details of potential participants with the researcher. Contact information of private practitioners was obtained online through websites of private hospitals, clinics and companies providing mental health care. All mental health professionals were invited to participate via email with information of the study attached. A two-week time allowance was given to reflect upon the invitation before a follow-up telephone call was made. Only upon this initial agreement did the researcher request for a time and place of interview most convenient to each participant. A written consent form was provided prior to commencement of each interview. Participation was on an unpaid voluntary basis.

To ensure quality, comfort and uninterrupted flow of the interviews, the sessions were held in settings that allowed complete privacy and confidentiality. External noise was kept to a minimum and other people or devices, such as mobile or office phones, did not interrupt sessions. All interviews were tape recorded and transcribed. Each session lasted approximately 45 minutes.

The interview questions were semi-structured and it evolved from the opening questions of ‘What is stigma of mental illness?’ and ‘How does it operate in the individual and social context?’. Subsequent questions were dictated by participant responses, however, the general pattern of discussions revolved around the extent and impact of stigma, forms of stigmatisation and/or discrimination and groups of people being stigmatised most. The interviews generally ended with the exploration of ideas towards strategies towards addressing stigma and discrimination.

Data was analysed using thematic analysis. This approach proposed by Merriam [34], Mays and Pope [35], and Braun and Clarke [36] aims to identify repetitive themes and patterns throughout the transcript and interpret data accurately from the participants’ perspectives. Due to the exploratory nature of the present study, thematic analysis was deemed the most appropriate, flexible and resourceful tool allowing for exhaustive, detailed and complex accounts to be generated from the data [36]. It draws themes and patterns exclusively from the data while not being theoretically confined. As this study was time-limited, thematic analysis was deemed most appropriate, as it does not involve testing of emerging themes or thematic saturation or require additional collection of data.

Reliability in this study is crucial. The researcher made a conscious effort to set aside pre-existing conceptions or expectations and put themselves in the position of the participant during the process of reading and coding all transcripts. This is to ensure data are interpretive epistemology [37].

Additionally, inter-rater reliability is desirable [34,36] to demonstrate the extent of consistency and agreement in data coding. Therefore, a volunteer from Malaysia with good qualitative experience was engaged in part of the data analysis.

The interviews were transcribed and transcripts read and coded by the researcher. After the initial coding and clustering of emergent themes, the relevance of the codes and consequently, themes were discussed with the qualitative research volunteer. Thereafter, as per Merriam [34], we engaged in an examination process once the sub-themes and themes were established whereby the researcher first shared individual interpretations of the data. The basis for such views were challenged until consensus on the most accurate interpretation of participants’ answers was achieved. This is to ensure the analysis is not merely interpretations based on personal ideas. Each transcript was re-visited by the main researcher and qualitative research volunteer to seek for evidence on respective views until all themes and sub-themes were agreed upon. Thus, inter-rater reliability is obtained.

## Results

In this study, all references made by the participants regarding stigma of mental illness referred to the past 1 to 10 years of their work experiences as mental health practitioners. Participants responded based on their direct observations of and reported experiences from their patients. Seven principle themes emerged from the thematic analysis of this study. Each of the themes encapsulates corresponding subthemes as demonstrated in Table 1 below.

**Table 1:** **Emerging themes and sub-themes**

**No**	**Theme**	**Sub-theme**
1	Perpetrators	1.1 Family
		1.2 Friends
		1.3 Employers
		1.4 Health-related alliances
2	Types of mental illness carrying stigma	2.1 Schizophrenia
		2.2 Bipolar disorder
		2.3 Depression
3	Demography and geography of stigma	3.1 Urban vs. rural
		3.2 Ethnicity
4	Manifestations of stigma	4.1 Labelling
		4.2 Rejection
		4.3 Employment
5	Impact of stigma	5.1 Individual
		5.2 Function in society
6	Causes of stigma	6.1 Lack of education and awareness
		6.2 Media portrayal
7	Proposed initiatives	7.1 Advocacy
		7.2 Policy and legislation

In general, it transpired that mental health professionals are aware of stigma and discrimination towards people with mental health problems. All participants agreed that the prevalence of the phenomenon in Malaysia is high. This indicates a significant concern of the possible entrapment in vicious cycles.

### Perpetrators

Alarmingly, all participants reported on key parties who often stigmatised against persons with mental health problems. We then classified this occurrence under the theme ‘perpetrators’. The four main groups of people who were mentioned to discriminate most towards people with mental health problems are: **‘family’, ‘friends’, ‘employers’,** and **‘health-related alliances’.**

#### Family

Family members, as perpetrators, generated almost full consensus (12 out of 15) amongst the mental health professionals as actively engaging in stigmatising and discriminating against their family members with mental illness. In this context, participants referred specifically to parents, siblings, in-laws, aunts, uncles and cousins as the main ‘perpetrating family members’. As quoted,

*“There have been cases when a patient is discharged (from hospital), no family members came to pick them up. So, we get the ambulance to send them back. But when they (family) see the patient coming home, they lock the doors and windows. Pretending like they are not home”. – [P004, government psychiatrist].*

#### Friends

Eight out of the 15 participants mentioned that their patients also complained about the difficulty in maintaining existing relationships of all kind but especially in making new friends.

*“Some friends are nice to you but the minute they know you’re mentally unstable, that’s when you notice they won’t answer your calls or don’t hang out with you anymore. It’s devastating for the client (patients)”. – [P002, government psychiatrist].*

One mental health professional raised an interesting explanation for such discriminating behaviour stating that,

*“Friends are scared of knowing about your illness maybe because they don’t want to be responsible if anything happens when they are with you. Mental disorder is unpredictable”. – [P012, private counsellor]*.

#### Employers

Almost half of the mental health professionals (7 out of 15) reported that their patients complained about active stigma and discrimination by their employers. The excerpts below indicate the alarming negative behaviours that people with mental health problems may face in the workplace.

*“Employers think you are a risk. It’s a challenge for my patients to disclose his or her condition especially during [job] interviews. There’s one case where my patient told the potential employers about his condition at the final stage of interview and they withdrew his offer”. – [P008, government psychiatrist].*

*“One patient told me that he took sick leave because he was depressed. Then, when he came back, he was told he is fired”. – [P013, government psychiatrist].*

#### Health-related alliances

Interestingly, despite their role in caring for patients, health workers such as nurses were found to label patients with derogatory terms such as ‘crazy’ and ‘nuts’. They also often undermined patients as having limited chances of recovering from their condition, quoting,

*“Because the staff think mentally ill people can never recover, they seem to pay less attention to their wellbeing. Sometimes when patients complain of physical illness, the staff can just ignore because they think the patients is acting out. It’s dangerous. Can even lead to death if serious enough”. – [P014, private clinical psychologist].*

Furthermore, a worthwhile finding was generated from two practitioner’s accounts on insurance companies. At present, insurance policies in Malaysia do not allow persons with mental illness to acquire health insurance nor does the coverage include psychiatric services. This creates potential problems, as patients tend to avoid seeking public mental health services due to the existing stigma. Whereas, the high cost of private healthcare often strains patients financially and may sometimes deter them from help-seeking all together.

It is also noteworthy to report that the participating health professionals, as service providers, were also victims of stigma. At least three participants claimed to have been avoided by the public due to their role and association in mental health services. However, this report was merely based on personal suspicion and not a matter that was officially filed to any authority.

### Types of mental illness carrying stigma

As the participating mental health professionals elaborated on their patients’ plight of being stigmatised and discriminated against, types of mental illness most likely to carry stigma came to light. Full consensus was achieved (15 out of 15) on **schizophrenia** being the mental health condition carrying most stigma and receiving most discrimination in Malaysia, according to their patients, followed by **bipolar disorder** and **depression**.

Half of the participants reported that the general public believes that personal dispositions such as bad genes and psychological weaknesses attribute to their patients’ conditions. Moreover, mental health professionals also cited other people’s supernatural beliefs as one of the criticisms plaguing patients. Participants further elaborated that stigma against patients with these illnesses occur mostly due to their symptoms being observable and unpredictable; hence schizophrenia being the most stigmatised condition. Mental health professionals quoted the following,

*“It is because they (patients with schizophrenia) seem to act weird and behave abnormally (when they’re experiencing an episode), and they’re unpredictable that people become afraid of them. People just don’t understand them”. – [P001, private psychiatrist].*

*“People believe that if you are depressed, it means you are weak”. – [P014, private clinical psychologist].*

### Demography and geography of stigma

This particular theme on demography and geography of stigma is characterised by the differences in the general public’s perceived level of tolerance towards mental illness according to their demographical and/or geographical make-up.

#### Urban vs. rural

It was found that people living in rural areas are more accepting of mental illnesses, attributing this to their strong collective community lifestyle (social capital) that acts as a protective factor.

*“People in the villages care for you more. Because they live in a close community. Not like the people here [Kuala Lumpur]”. – [P011, government psychiatrist].*

To further elaborate, P004, a government psychiatrist, quoted a patient saying,

*“Orang kampung (people living in rural areas), as long as you don’t bother or affect them, it (mental illness) doesn’t matter to them”.*

In stark contrast to this, urban living is considered to be a risk factor for stigma and discrimination of mental illness. This is possibly due to urban settings having better health care access and resources; as such, individuals are expected to manage themselves without relying on societal support. As mentioned by P007, a private clinical psychologist,

“*It’s tough being a mental patient in Kuala Lumpur (a city) because people don’t support you. They discriminate or avoid you”.*

#### Ethnicity

Additionally, a third of mental health professionals linked ethnicity to higher or lower stigma of mental illness. Three main ethnic groups - Malay, Chinese and Indian - make up the majority of the Malaysian population. It was reported that the ethnic Malay population is more likely to believe that mental illness stems from supernatural activities and thus are highly likely to reject the condition. As demonstrated by the following example:

*“Orang Melayu (the Malay people) tend to believe my schizophrenia patients are possessed by supernatural beings”.* - [P003, a private clinical psychologist]

Whereas, for reasons yet to be understood, Chinese families were reported to be more accepting of mental illness whereby P004, a government psychiatrist, cited that:

“*The Chinese community is now getting more aware about mental health. In a popular Chinese radio channel, they actually have public announcement for people to seek professional help if they feel depressed”.*

However, none of the mental health professionals mentioned, either at individual or community level, stigma and discrimination of mental illness by the Indian ethnic group in Malaysia.

### Manifestations of stigma

From the coding of the interviews, it emerged that all participants concurred that **labelling** and **rejection** in all areas of life as well as active **employment** discrimination are the three most common occurring manifestations of stigma.

#### Labelling

Labelling is the most complex, as it plays an important clinical role in understanding diagnosis, treatment and even research. Yet, all participants (15 out of 15) reported that within the social context, labelling is possibly the worst form of stigma patients confront.

*“My clients’ worst nightmares are when people characterise them according to their diagnoses”. – [P002, government psychiatrist].*

*“Name calling. That’s what my patients are afraid of. They can accept their condition but other people don’t. People judge”. – [P010, private psychiatrist].*

#### Rejection

Similarly, the interviews showed full consensus on rejection – and subsequent social exclusion – as a key way of stigmatising people with mental illness. Family and friends appeared to be the main perpetrators in this sense.

*“They believe that if something is wrong with the patient, there’s something wrong with their genes so that is why they feel the need to ‘expel’ the patient from the family. So who is to care for them when their own family won’t?” – [P002, government psychiatrist]*.

*“Friends don’t understand what is happening to you so when you’re sick they [friends] just don’t want you around. Because you act weird or abnormally. It’s unfair to them [patients]”. – [P009, private psychiatrist].*

#### Employment discrimination

In addition to labelling and rejection, employment-related discrimination is the next most common form of actively stigmatising and shunning people with mental illness from key areas of life. Half of the participants stressed the lack of employment prospects due to active employment discrimination as debilitating for patients as it renders them without any platform for independence, dignity and participation in wider civil society and the economy. One mental health professional stated that:

*“I don’t understand why they [employers] can’t just give simple jobs, such as cleaning, to the patients. What’s so difficult about sweeping or mopping that they can’t do? And how do you expect them to have a stable life if you don’t give the opportunity?” – [P008, government psychiatrist].*

### Impact of stigma

In discussing the impact of stigma on people with mental health problems, several issues came to light in the interviews. The two main consequences of stigma are its impact on the **‘individual’** itself as well as their **‘function in society’**.

#### Individual

According to more than half of the mental health professionals (8 out of 15), their patients’ self-perception and self-empowerment are most adversely impacted by stigma. These may consequently affect patient’s help-seeking behaviour and compromising their recovery.

*“It’s a vicious cycle. People avoid or reject them. Then they feel neglected and they feel small. So they refuse to come to the clinic or hospital because people will see them there. Obviously, without treatment, they are going to get worse and what happens next? More stigma. So who’s going to break the cycle?” – [P008, government psychiatrist].*

#### Function in society

As a result of stigma at an individual level (self-stigma), the effect may extend to how patients’ are able to function within society. When rejected by their family, friends, current and prospective employers, patients often end up homeless thus restricting their role as a contributing member to wider society. Whereas, due to their potential and/or perceived need to go off sick more than others, employers often view patients as a liability to the organisation’s productivity.

Five of the participating mental health professionals explained how the inability for patients to be employed leads to incapability to be independent and to disempowerment with negative consequences not only for the individual but also for wider society.

*“Patients are thrown out of their own homes and they don’t know where to go. So they sleep by the road. Or at back alleys. They are left tattered and dirty. So how to get a job? Without a job, how to get money to live? So they are stuck, having to rely on people for it, sometimes having to beg. They have no power at all”. – [P003, private clinical psychologist].*

### Causes of stigma

In correspondence with the previous themes, all 15 mental health professionals reported that the actual causes of stigma of mental illness require serious attention in order to ensure that we not only thoroughly understand the causes but also properly, effectively and efficiently address them. From all the responses, an all-around **lack of education and awareness** as well as negative **media portrayals** were referred to as the two of the biggest causes of stigma of mental illness and main concerns to be addressed.

#### Lack of education and awareness

Mental health professionals identified the lack of education and awareness surrounding mental health and illness as the biggest concerns and key areas to address. Mental illness involves a multitude of symptoms: from behavioural to mood changes. As reported, culture and religion play an important role as, on the whole, the general public in Malaysia believes in supernatural or spiritual causes as the aetiology of psychiatric conditions. This leads them to seek alternative healers as primary treatment before reaching out to the officially trained and recognised mental health professionals (both in public and private health services). In this case, traditional or alternative healers in Malaysia are generally without proper knowledge of mental health issues. Hence, resorting to such parties can be detrimental for conditions that may otherwise require medical attention. However, within Malaysian society, superstitious reasons are easier accepted than medical or physiological explanations due to the latter reflecting possible genetics or biological failure.

#### Media portrayal

Moreover – and in line with most parts of the rest of the world – mass media (including social media) is becoming the main avenue of information dissemination in Malaysia too. With it often comes an inaccurate portrayal of people with mental illness that has become a significant factor to stigma and discrimination of mental illness. Nine out of 15 participants believe that the media should take their responsibility for disseminating accurate and educational/awareness raising information surrounding wider mental health and wellbeing and therefore the media should be made one the main target groups for mental health advocacy and awareness-raising interventions, according to the mental health professionals.

### Proposed initiatives against stigma in Malaysia

With stigma of mental illness demonstrating to be increasingly debilitating for people with mental health problems, initiatives should be taken to deter further complications. All 15 participants agreed that wider **advocacy** and also the government’s role in **policy-making and legislation** are central in addressing stigma of mental illness.

#### Wider advocacy

Initiatives against stigma may include various parties and different approaches. However, all participants agreed on education and awareness-raising to be the most important whereby 9 out of 15 mental health professionals recommended mass media as a tool and 8 out of 15 proposed non-governmental organisations (NGOs) as key to mental health advocacy. Participants believe that education and awareness-raising are required to enable understanding and achieve higher tolerance and acceptance of mental illness in wider society. As part of the causes of stigma associated to media portrayal of mental illness, mental health professionals also emphasised educating individuals within and through mass media industry as one of the key strategies in addressing stigma of mental illness. As most of the initiatives are government-led, NGOs are able to complement these programmes through close collaboration and support for such activities.

#### Policy and legislation

Aside from advocacy, the law and policy are significant instruments in addressing stigma towards mental illness. In Malaysia, the Mental Health Act 2001, Mental Health Operational Services Policy 2011 and National Mental Health Policy 2013 are the current operational legislation and policies respectively. Although all participants agreed that policymakers are heading in the right direction, there are still many limitations that require attention.

## Discussion

### Stigma: who, how and why?

Drawing upon the themes that emerged from the analysis of the interviews, three main questions can be answered in relation to stigma of mental illness in Malaysia: *who*, *how* and *what,* and grouped accordingly. Firstly, the question *‘who’* refers to the *perpetrators*, the *demography of stigma*, and the *types of mental illness carrying stigma.* Whereas *‘how’* reflects on the stigmatising behaviours (*manifestations of stigma* and *impact of stigma)*. Finally, the question *‘why’* relates to the *causes of stigma*. From observation and reporting, stigma occurs as a vicious cycle whereby the different components (*who*, *how*, *why*) contribute to this system and are represented by these seven themes.

The outcome of family, friends and employers being described as the most discriminating parties reinforces findings of other research. Mubarak et al. [24], Lasalvia et al. [26,27], Thornicroft et al. [25] and Rose et al. [28] have all demonstrated similar patterns and reports of experienced discrimination. It is, however, a major concern as these main named *perpetrators* are exactly the people who usually have (or are in a position to do so) the most meaningful, trustful and influential relationships with the person experiencing mental health problems. Friends and particularly family are the main source of support, with values and beliefs dynamically shared amongst members of the group giving patients a sense of belonging and trust. With our daily activities mostly revolving around the home, social and workplace settings, stigmatising attitudes and discriminatory acts by the key people we interact with in these settings (such as family, friends, employers and colleagues) can instigate perpetual feelings of hopelessness, rejection, distrust, social exclusion and even total isolation.

People with psychiatric conditions often face a challenging time having to deal with their condition and are left even more vulnerable when the people closest to them or most significant in their lives engage in *stigmatising behaviours* which exacerbates patients’ negative perceptions of themselves. In this study, the participating mental health professionals cited decreased self-esteem and lack of empowerment as the two most common outcomes of discriminatory attitudes. These feelings reflect the *impact at an individual level* and failure to address these issues can in turn lead to wider disempowerment, incapacity and failure to *function within the society*. In line with results of Mubarak et al. [24], this study found stigmatisation taking a toll on patients’ personal independence, social relationships and employability, with Thornicroft et al. [25], Lasalvia et al. [26,27], Rose et al. [28] and Lauber and Rossler [19] yielding similar results. These disadvantages may be the consequence of patients being perceived as unable to care for themselves, unpredictable and even aggressive [21,22]. However, by limiting patients from these opportunities, stigma is actually reinforced. Accordingly, efforts to transform both mental health policy and care provisions in Malaysia should firmly focus on changing stigmatising behaviours and negative public perceptions of mental health and people with mental illness.

The impact of stigma was found to be most profound and outspoken in patients with a diagnosis of schizophrenia, bipolar disorder and depression, further validating Mubarak et al. [24], Thornicroft et al. [25], Lasalvia et al. [26,27] and Rose et al. [28]. These outcomes are not surprising as Corrigan [13] and Penn et al. [38] suggested stigmatising attitudes arising from the observable cues of these conditions (both in the private and public sphere). This was also reiterated in this study by the participating mental health professionals who cited evident symptoms and unpredictability as factors for instigating stigma in relation to these three disorders. Such evidence was also found in Chang and Horrocks [21].

Observations within this theme have also demonstrated perceptions of mental illness arising from what has been referred to as ‘personal weakness’ or ‘supernatural activities’. The Malay community especially has been cited as the group that holds most strongly to this belief, consistent to that discovered by Ng [20]. This poses an interesting debate as Fabrega [18] found Muslims in Malaysia to be supportive of mental health patients, attributing psychiatric disorder as naturally occurring and not associated to any moral meaning. In fact, Muslims believe they are responsible for people with mental health issues hereby failure to do so is seen as defying God’s will. With the Malaysian Malays being predominantly Muslims, their beliefs of mental illness do not correspond to those of Islam. This is confounding and further research may be useful in understanding this discrepancy and contextualise it. It also highlights the role of cultural influence on religious practice thus anti-stigma efforts in Malaysia need to take this notion into consideration. Conversely, although the Chinese community in Malaysia has been found to consistently perceive mental illness as shameful and a disgrace [18,20,21], this was not true in the present study as participants reported the Malaysian Chinese society as vigorously advocating for mental health. A concrete example of their initiative is to give public service announcement on a popular Chinese radio channel.

It is desirable that a universal approach to address stigma and discrimination be applied across the ethnic groups in Malaysia. However, this theme also highlights that cultural and ethnic differences need to be taken into account for anti-stigma interventions and campaigns, both within as well as across countries, as evidenced by the work of Knifton et al. [39] and Lasalvia and colleagues [27]. Understanding social group norms, values and beliefs is very crucial for contextualisation and tailoring needs-based interventions and campaigns suitable to each context. Therefore, this insight serves as a good platform to understand how best stigma of mental illness can be tackled across the country effectively, both as a whole as well as to different target groups. Furthermore, social capital was found to be a protective factor against stigma [40] and societies or certain ethnic groups with higher social capital and better social cohesion seemed to respond better to anti-stigma interventions than very individualised societies with lower social capital and social cohesion [27]. This would explain why certain anti-stigma campaigns or interventions may work in one particular context but not in another.

Special attention should also be given to discriminating behaviours from health staff against patients. Although the number is small, this report affirms the outcome of Minas et al. [23] that stigmatising attitudes amongst health care workers towards patients are predominant. This is a serious issue as stigmatising behaviour from mental health professionals towards their patients jeopardises the patient-practitioner relationship, trust, adherence to a treatment plan, and subsequent recovery whilst at the same time not only negatively impacting on a person’s life and wellbeing but also on mental health services and the wider health system in Malaysia. In line with Jamaiyah [31], Mubarak [32] and Haque [33] governance of mental health services in Malaysia is necessary, if not critical. Better training and awareness-raising workshops can be utilised as means of education amongst health staff.

Insurance companies too, as cited by participants, play a role in contributing to the debilitating life of a mental health patient. Insurance is an important component in matters such as health and housing. With limited eligibility for insurance, people with mental health problems are yet again left battling the vicious cycle of discrimination and disempowerment. Especially in Malaysia, private healthcare seems a more desirable option for mental illness treatment due to its perceived privacy (and privacy is desired due to fear of stigma). However, without insurance, patients may resort to minimising treatment or even opting out of it. As a result, quite a number of patients do not seek help, are left untreated, and thus reinforcement of stigma occurs.

Interestingly, participating mental health professionals interviewed in this study expressed that they too experienced stigmatisation due to their association to mental ill health and people with mental health problems. Despite not being debilitating, this may still leave an undesirable impression on the field of mental health and mental health practice in Malaysia and, consequently, deterring future generations of good mental health practitioners from embracing their profession. This is an indication of the extent of mental health misconception in Malaysia, which urgently requires mental health advocacy, changes in policy and practice, raising education and awareness, and capacity building in both civil society and the wider health system, as reported by the participating health professionals in this study.

As Thornicroft et al. [3] argued, stigma in mental health is a consequence of problems of knowledge (ignorance); problems of attitudes (prejudice); and problems of behaviour (discrimination). This rings true for the findings of this study too. Furthermore, Khan et al. [22] affirmed that full consensus was achieved on lack of awareness and education as the main *cause of stigma* in Malaysia. This is followed by negative portrayal of psychiatric conditions in the media that may fundamentally stem from the limited awareness and lack of education. In hindsight, the Malaysian society’s beliefs of mental health are representative of the problems suggested by Thornicroft et al. [3]. Especially in our current media-driven society, mental health advocacy and education can be disseminated through various forms especially social media. Ultimately, education is key to engaging public’s awareness on mental health and thus contributing towards the eradicating of stigma of mental ill health.

### Proposed initiatives from service providers’ viewpoint

In line with Jamaiyah [31], Mubarak [32], Haque [33] and Lauber and Rossler [19] the participating mental health professionals in our Malaysian study expressed that anti-stigma initiatives, advocacy, policy and legislation are suggested as vital and most influential means of addressing stigma in civil society and the health system. This result is unsurprising as a lack of education and awareness was deemed to be the main cause of stigma in Malaysia. Mental health professionals exclaimed to be especially keen on more non-governmental organisations (NGOs) to actively engage in advocacy of mental health issues. This is to enable wider outreach to the public. For instance, different NGOs can address specific areas of mental health, to various target groups and through diverse media. Thus, greater dissemination of information to wider audiences in society can be achieved.

On the other hand, the interviewed service providers believed that the current mental health policy and legislation are inadequately implemented. One of the most alarming issues raised in this study is the poor governance of non-medical mental health service providers. The current Mental Health Act 2001 and Psychiatric and Mental Health Operational Services Policy 2011 are predominantly applicable to government psychiatrists. Licensing and code of conduct does not exist for clinical psychologists in Malaysia. Such freedom to practice puts patients’ wellbeing at stake, as quality of services is not monitored. Failure of mental health care quality management in itself is a form of discrimination.

### Strengths and limitations of the present study and recommendation for future research

The present study is innovative in a few key aspects. First of all, the paper approached stigma of mental illness from a wider perspective rather than from a specific disorder. As such, mental health programmes can be tailored to suit the most pressing needs of overall mental health care. Subsequently, mental health practitioners were engaged. This enabled the viewpoint on stigma to be more objective and, perhaps, less likely emotionally influenced. Most importantly, this study highlights the very pressing needs of improvement of mental health services especially in relation to needs-based policies, practices and proper implementation and evaluation in Malaysia.

However, the study is not without its drawbacks. As participants were all mental health service providers practising within urban demographic settings, the emerged study findings only apply to these settings in Malaysia. Another limitation is the lack of insight into the Indian community as part of the major ethnic groups in Malaysia. Further in-depth qualitative research addressing other geographical areas (rural versus urban) and insights from all different ethnic groups in Malaysian society would be most helpful in formulating solutions that are not only tailored to the Malaysian context as a whole but also to the more specific geographical, demographical, and ethno-cultural needs to improve mental health services and combat mental illness stigma in Malaysia.

In addressing these gaps, future research is advised to include stakeholder groups in society and the health system such as patients, carers and policymakers. Investigating participants from rural areas is also desirable. Especially with the existing implementation of community mental health care in Malaysia [31-33,41], investigating the extent of stigma within rural society is beneficial as the health care system may be tailored to suit both, urban and rural communities. Furthermore, dissecting the cultural background and examining the differences between ethnic groups in Malaysia may yield better insight into stigma in the country. Ultimately, a review of the strengths and limitations of our current policy and practice is highly useful.

## Conclusion

Drawing upon the results of this study, stigma of mental illness and of people with mental health problems was found to be a profound phenomenon in Malaysia. Specifically, patients with a diagnosis of schizophrenia, bipolar disorder and depression were highlighted to be the ones receiving most stigma and discrimination and therefore need pressing attention. Family, friends and workplace staff were mentioned to be the main and most discriminating groups. The personal and societal implications of stigma found patients to be disempowered, socially excluded and trapped in a vicious cycle of discrimination. Thus, there is a pressing need to address stigma of mental illness in civil society and the health system especially, as stressed by the mental health professionals in this study, through education and awareness raising campaigns taking into consideration the multi-cultural background of Malaysia. Further in-depth qualitative research with all stakeholder groups in society and the health system is required in order to effectively, efficiently and sustainably develop interventions to tackle stigma and discrimination of mental health and people with mental illness in Malaysia.
